# The complete mitochondrial genome of *Porites harrisoni* (Cnidaria: Scleractinia) obtained using next-generation sequencing

**DOI:** 10.1080/23802359.2018.1443852

**Published:** 2018-02-24

**Authors:** Tullia Isotta Terraneo, Roberto Arrigoni, Francesca Benzoni, Zac H. Forsman, Michael L. Berumen

**Affiliations:** a Red Sea Research Center, Division of Biological and Environmental Science and Engineering, King Abdullah University of Science and Technology, Thuwal, Saudi Arabia;; b ARC Centre of Excellence for Coral Reef Studies, James Cook University, Townsville, Australia;; c Department of Biotechnologies and Bioscience, University of Milano-Bicocca, Milan, Italy;; d Hawaii Institute of Marine Biology, Kaneohe, HI, USA

**Keywords:** Mitogenome, Scleractiania, ezRAD sequencing

## Abstract

In this study, we sequenced the complete mitochondrial genome of *Porites harrisoni* using ezRAD and Illumina technology. Genome length consisted of 18,630 bp, with a base composition of 25.92% A, 13.28% T, 23.06% G, and 37.73% C. Consistent with other hard corals, *P. harrisoni* mitogenome was arranged in 13 protein-coding genes, 2 rRNA, and 2 tRNA genes. *nad5* and *cox1* contained embedded Group I Introns of 11,133 bp and 965 bp, respectively.

Corals of the genus *Porites* are among the most ecologically common corals in the tropics (Veron 2000). In addition to contributing to the fundamental framework for many coral reefs, corals in the genus *Porites* are among the most resilient to mass bleaching events, which are projected to increase under future climate conditions (Baker et al. 2004). *Porites harrisoni* (Veron 2000) is a major framework builder in some of the hottest seas in the world, such as the Red Sea and the Persian Arabian Gulf (Davis et al. 2011; Roik et al. 2016). Understanding the mechanisms shaping corals resistance in such extreme environments is crucial for predicting future reef scenarios (Voolstra et al. 2015).

Here, we sequenced the complete mitochondrial genome of *P. harrisoni* (GenBank accession number MG754070) using restriction site associated DNA (RAD) sequencing. The coral sample used in this study was collected at Dhi Dahaya Reef in the southern Saudi Arabian Red Sea (N 16°52.377′, E 41°26.409′), deposited at King Abdullah University of Science and Technology, Saudi Arabia (voucher number SA1704), and the coral tissue was preserved in 98% EtOH. DNA extraction was performed with DNeasy^®^ Blood and Tissue Kit (Qiagen Inc., Hilden, Germany) and quantification was performed with Qubit 2.0 fluorometer (Invitrogen, Carlsbad, CA). We used ezRAD sequencing method, and MboI and Sau3AI (New England Biolabs, Ipswich, MA) frequent cutters. ezRAD libraries have been prepared with TruSeq^®^ Nano DNA kit, and paired-end sequenced with the HiSeq^®^ 4000 platform in the Bioscience Core Lab facility at King Abdullah University of Science and Technology, Saudi Arabia. The obtained reads were assembled to the deposited reference mitogenome of *Porites lobata* (NC030186) using Geneious^®^ v.10.1.3 (Biomatters Ltd. Auckland, New Zeland). A 0% majority option for coverage greater than 3× was applied when calling a consensus sequence, which assembled to 95.2% of *P. lobata* genome. Genes were annotated with DOGMA (Wyman et al. 2004) and MITOS (Bernt et al. 2013), and manually verified.

The complete mitogenome consisted of 18,630 bp, with a base composition of 25.92% A, 13.28% T, 23.06% G, and 37.73% C. The total number of protein-coding genes was 13, consistent with the typical scleractinian mitogenome organization (Medina et al. 2006; Fukami et al. 2007). A total of two ribosomal RNA (*rnl, nrs*) and two transfer RNA (*trnM*, *trnW*) genes was recovered. Among the 13 protein-coding genes, *nad5* and *cox1* were interrupted by Group I Introns, 11,133 bp and 965 bp in length, respectively.

*Porites harrisoni* phylogenetic position with reference to other Scleractinia has been reconstructed using Bayesian inference as implemented in MrBayes 3.1.2 (Ronquist and Huelsenbeck 2003) for 4,000,000 generations and maximum-likelihood as implemented in PhyML 3.0 (Guindon et al. 2003) with 1000 bootstrap replicates. The mitogenomes phylogeny shows that all *Porites* sequences, including *P. harrisoni* cluster in a single group (Figure 1).

**Figure 1.**
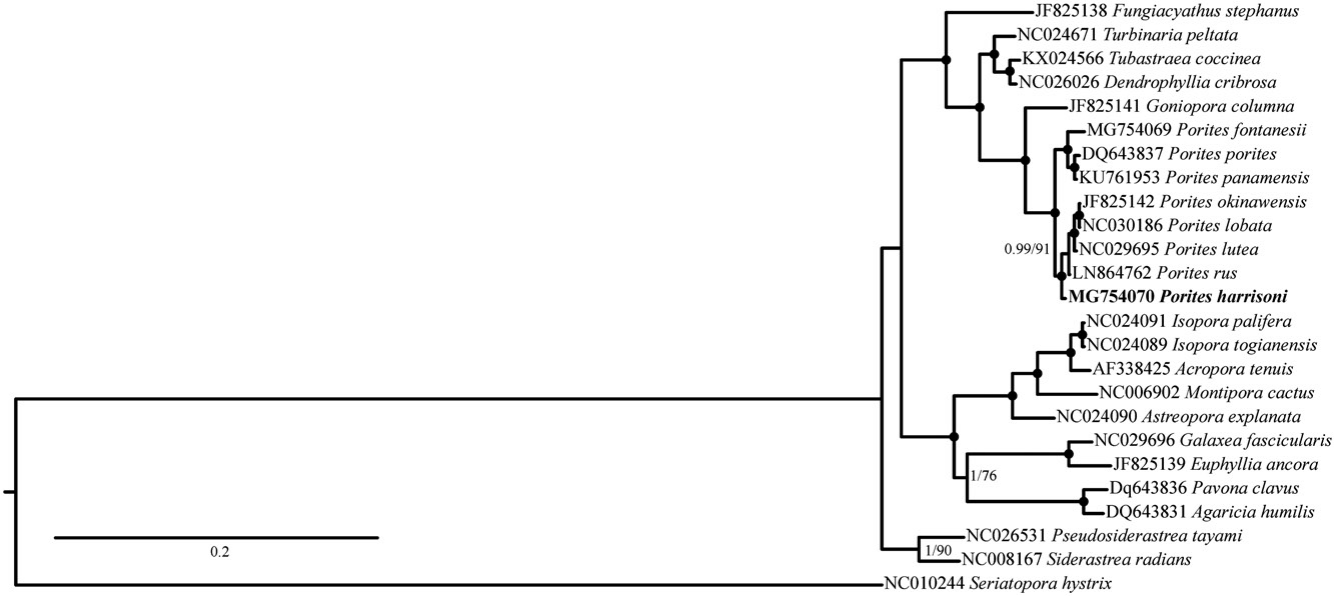
Phylogenetic reconstruction based on complete mitochondrial genomes of *P. harrisoni* (in bold) and other Complex scleractinian corals. Numbers at nodes represent Bayesian posterior probabilities (Bp) and maximum likelihood bootstrap (ML) values. Black dots represent Bp = 1 and ML = 100. *Seriatopora hystrix* was selected as outgroup.

The complete mitochondrial genome of *P. harrisoni* will provide a baseline for further studies aimed at a better understanding of the evolution and adaptation of Scleractinia.
